# CAVE: An Open-Source Tool for Combined Analysis of Head-Mounted Calcium Imaging and Behavior in MATLAB

**DOI:** 10.3389/fnins.2018.00958

**Published:** 2018-12-18

**Authors:** Jennifer Tegtmeier, Marcel Brosch, Kathrin Janitzky, Hans-Jochen Heinze, Frank W. Ohl, Michael T. Lippert

**Affiliations:** ^1^Systems Physiology of Learning, Leibniz Institute for Neurobiology, Magdeburg, Germany; ^2^Department of Neurology, University of Magdeburg, Magdeburg, Germany; ^3^Center for Behavioral Brain Sciences (CBBS), Magdeburg, Germany; ^4^Behavioral Neurology, Leibniz Institute for Neurobiology, Magdeburg, Germany; ^5^Faculty for Natural Sciences, Otto-von-Guericke University Magdeburg, Magdeburg, Germany

**Keywords:** single-photon head-mounted calcium imaging, *in vivo* imaging, freely moving, awake behaving, open source software, image analysis

## Abstract

Calcium imaging in freely behaving rodents using head-mounted miniature microscopes is currently becoming an increasingly popular technique in neuroscience. Due to the large amounts of complex data that the technique produces, user friendly software is needed for quick and efficient processing. Here, we present a new tool for analyzing calcium imaging data from head-mounted microscopes together with simultaneously acquired behavioral data: CAVE (Calcium ActiVity Explorer). CAVE bundles a unique set of algorithms specifically tailored to the analysis of single-photon imaging data from awake behaving animals including efficient motion correction and automatic ROI selection with manual audit and refinement. For behavioral analysis, CAVE can automatically track animal position and orientation. Individual behavioral epochs and external events can then be analyzed in correlation to calcium imaging and tracking data. Our program is written in MATLAB, the source code is open source and particularly focuses on providing a streamlined workflow for novice users while also retaining detailed configuration options for advanced users. We evaluate the performance of CAVE by investigating neural activity in hippocampus and somatosensory cortex. The fast analysis provided by CAVE allowed us to track activity in a large set of animals over the course of several months during exploration behavior, detailing the properties of onset and offset of observable activity and the visible cells per imaging location.

## Introduction

Head-mounted imaging is currently rapidly growing in popularity due to the development of several lightweight microscopes which allow imaging in freely behaving mice (Ghosh et al., 2011; Cai et al., 2016; Francis et al., 2017). Such imaging can simultaneously capture the activity of hundreds to thousands of cells. While the processing of two-photon imaging data has reached a high level of maturity, processing of single-photon imaging data poses different challenges. For example, no depth information is available, individual cells are not as clearly delineated and might overlap, image contrast is low, computer memory requirements are high, and movement artifacts are common.

Previous approaches have already addressed some of these challenges, thereby resulting in a small number of tools (Table 1) to analyze such data (Ghosh et al., 2011; Cai et al., 2016; Giovannucci et al., 2018; Zhou et al., 2018). What is currently lacking is a tool to analyze calcium imaging data in combination with simultaneously recorded behavioral data – the possibility for obtaining such combined data sets being a key strength of head-mounted microscopes in the first place. In addition, many tools are not particularly suited for the low-contrast, artifact plagued data from head mounted microscopes, cumbersome to use for novice users and might not even provide a graphical user interface (GUI). However, since large datasets are quickly acquired for several subjects and across dozens of sessions, ease-of-use is a critical factor determining the usefulness of the software and quality of resulting analyses. For example, due to overlapping cells, neuropil signal or sparse activity, cells might not always be correctly identified automatically and manual validation of automatic processing becomes a necessity to ensure data quality.

**Table 1:** Feature overview of available head-mounted calcium imaging analysis tools.

	Open source	GUI	Behavioral detection	Processing across sessions	Advanced settings	Reference
CaImAn	Yes	Limited	Limited	Yes	Yes	Giovannucci et al., 2018
Inscopix mosaic	No	Yes	No	Yes	Yes	Commercial
Miniscope analysis	Yes	No	Limited	Yes	Yes	Cai et al., 2016
DoricStudio	No	Yes	No	No	Limited	Commercial
CAVE	Yes	Yes	Yes	No	Yes	–

To address these needs, CAVE provides the user with a set of analysis functions particularly tailored for head-mounted single-photon imaging, such as motion correction of low contrast images, artifact removal, flat field correction in an intuitive GUI with context sensitive help messages, manual correction of automated cell detection, and integrated behavioral analysis. Instead of providing a myriad of different analysis options, we implemented only those methods – and optimized their parameters – into CAVE, which we found to be robust for single-photon imaging data from different brain regions.

The streamlined calcium data analysis workflow is composed of the following five steps: pre-processing, motion correction, Δ*F*/*F* calculation, cell detection, and calcium trace analyses. Behavioral analysis is composed of animal tracking, region of interest analysis, and behavioral tracking. Neural activity can then be correlated with the position of the animal. Flexible behavioral tracking lets the user define up to eight different behaviors and split neural activity accordingly.

## Methods

### Animals

For imaging, GP5.Thy1(GCaMP6f) mice were used, housed at 21°C and 40–60% humidity under a 12 h light–dark cycle. Food and water was provided *ad libitum*. All experiments were conducted according to the guidelines of the European Community (EUVD 6 86/609/EEC) and approved by a local ethics commission of the State of Sachsen-Anhalt.

### Surgery

The animal was anesthetized with Pentobarbital (50 mg/kg) and placed on a heating pad in a digital stereotaxic frame (DigiW, Neurostar). Dexamethasone (0.1 mg/kg) was given subcutaneously to prevent brain swelling. Before incision, bupivacaine (0.25 %) was subcutaneously injected to provide local anesthesia of the surgery site. Subsequently, the skin was incised with a scalpel and the periost removed. A tight cross hatch pattern was cut into the skull with the scalpel blade and tissue glue (Vetbond, 3M) applied to facilitate implant stability. For lens implantation in somatosensory cortex, a hole was drilled at the following coordinates AP: −1.0, ML: 0.7, and for hippocampus at AP: −2.1, ML: 1.7. For imaging in somatosensory cortex a prism-lens probe was implanted at a depth of 1 mm according to the size of the prism. For imaging hippocampus, regular lens cannulas of 3.1 mm length were implanted at a depth of −1.9 mm. The cannula was then fixed to the skull with dental acrylic.

### Head-Mounted Calcium Imaging

**FIGURE 1:**
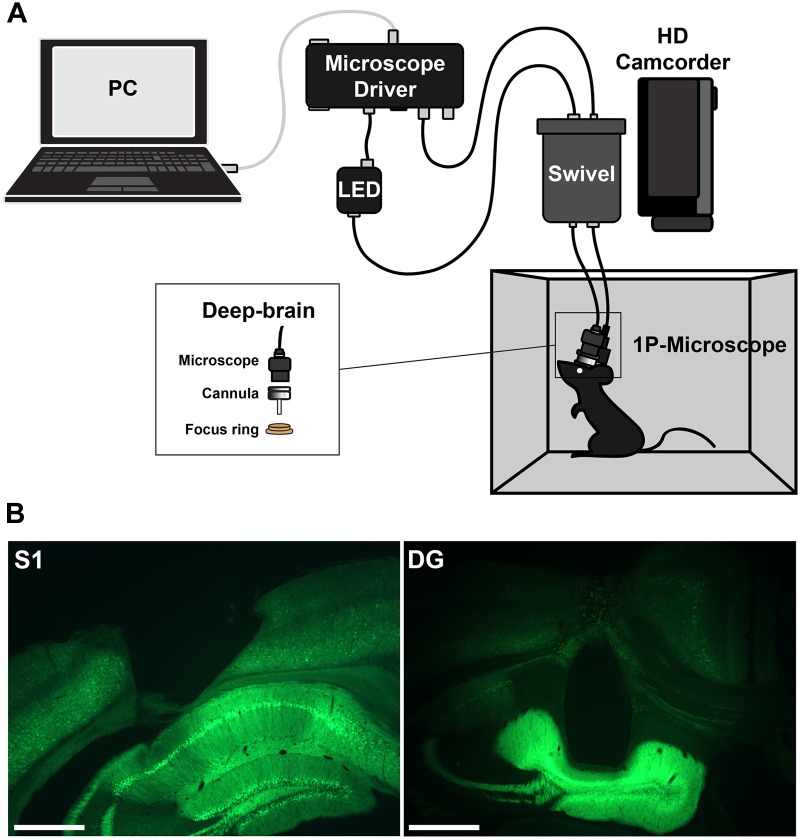
**(A)** Setup for head-mounted calcium imaging in freely moving animals. **(B)** Histological slices showing the location of the imaging prism probe in somatosensory cortex (left) or regular cannula in hippocampus (right) and the expression of GCaMP6f (green).

Imaging was performed with a head-mounted calcium imaging system (deep-brain system, Doric Lenses). An overview of the setup is shown in Figure 1. Behavior videos for tracking were acquired with a commercial HD camcorder (Panasonic HC-V160).

Experiments started 1 week after surgery. To enhance video tracking of the animal, each mouse was marked with color markers. In each session, the mouse was given two minutes to explore a small box with five different objects, each object in a different corner and one in the middle. If no cell activity was visible, the mouse was imaged once a week. Once cell activity started to emerge, imaging was done every 2–3 days initially with longer intervals later. Once visible cell activity had subsided the animal was perfused.

### Histology

Correct placement of the cannula lenses was confirmed histologically (Figure 1). Brains were freeze-sectioned into 60-μm slices using a cryotome, mounted in Mowiol, and imaged using an upright fluorescence microscope (Axioskop 2, Carl Zeiss).

### Data Analysis

Statistical analysis was performed in Prism (GraphPad). Normality was assessed with the Shapiro–Wilk normality test and appropriate non-parametric tests performed according to the type of comparison, as indicated with the respective result.

### Software Performance Analysis

All performance tests were conducted using a desktop PC with Intel^®^Core^TM^ i7-3770 processor, 16 GB RAM and Windows 10 64-bit installed. We used DoricStudio version 4.0.4.18, Mosaic version 1.2.0, Miniscope Analysis GitHub version from 17.03.2017, and Suite2P GitHub version from 17.10.2018. We chose five data sets of the same mouse expressing GCaMP6f that had been recorded in the dentate gyrus (DG).

One data set was chosen for comparing the different alignment algorithms of the toolboxes. This dataset showed a slight rotation and noticeable shaking of the image. To align the images, we used the regional Lucas Kanade algorithm of CAVE, the default subpixel registration algorithm of DoricStudio, the regional subpixel registration of Mosaic, regional FFT image registration algorithm of Miniscope Analysis, and phase correlation algorithm of Suite2P. CAVE, Mosaic, and Miniscope Analysis allow selecting a seed region for alignment; therefore, we selected the same 40 × 40 pixel area of the data set. For Miniscope Analysis, we converted the TIFF file into an uncompressed AVI file. Suite2P is particularly geared toward two-photon analysis; hence, spatial high pass filtering was necessary to get accurate alignment. All aligned image series were then processed in the same manner: first, we applied flat field correction by dividing each image by the heavily blurred mean image of the series; second, we cropped the image series to the middle 80%; third, we normalized the images to range between 0 and 1. Then, we calculated the mean image of the series and calculated Pearson’s correlation coefficient of each image to the mean image. Another four data of ascending size were chosen for runtime analysis: 1, 2.4, 4.5, and 9.5 GB. Due to minimal cropping of high-pass filter edge artifacts, the data sets for Suite2P became smaller: 0.7, 1.6, 2.7, and 4.0 GB. For Miniscope Analysis, data set size increased to the following values due to AVI conversion: 1.5, 3.6, 6.8, and 14.2 GB. Total processing time covers only calculation time, not the manual efforts of the user. For each software, data were loaded, pre-processed, aligned, and cells detected. During this whole process, peak memory was tracked.

## Architecture of CAVE

### Software Requirements and Availability

Calcium ActiVity Explorer was optimized to work with MATLAB version 9.1, Image Processing Toolbox version 9.5, Signal Processing Toolbox version 7.3, and Statistics and Machine Learning Toolbox version 11. CAVE is available from the authors and on Github: https://github.com/mtlippert/CAVE. While MATLAB is not open source, we decided to use it over a full open source toolchain due to the wide adoption in the neuroscience field and the fact that many students are already familiar with it.

### Functionality

**FIGURE 2:**
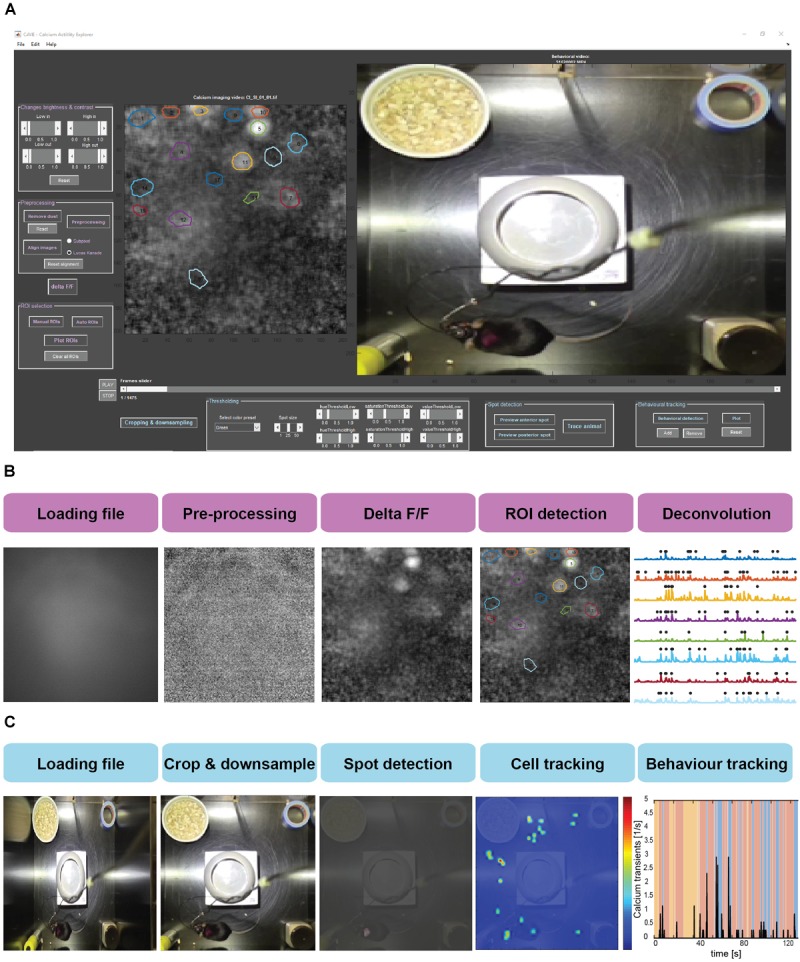
**(A)** Screenshot of the CAVE user interface. **(B)** Workflow for processing calcium imaging data. **(C)** Workflow for processing behavioral data.

Data processing in CAVE is divided into several steps summarized in Figure 2.

#### Dust Removal and Pre-processing

Dust on one of the many optical surfaces is a frequent occurrence in head-mounted calcium imaging experiments. The dust removal algorithm is capable of removing any kind of such unwanted regional abnormality in the calcium imaging video, as long as the abnormality is relatively stationary. This feature critically improves subsequent flat field correction and image alignment. Algorithmically, CAVE calculates the average intensity of a ring surrounding the selected area with eight pixels width and fills the area with this value. Further pre-processing includes video down-sampling, correcting for faulty frames, and flat field correction. Depending on the used image sensor in the camera, the resolution of head-mounted calcium imaging systems often vastly oversamples the spatial resolution provided by the sample and lens. Therefore, the time series is first down-sampled if it is wider than 100 pixels to increase processing speed (default parameter: 40% size). A further feature of long video sequences, captured with a high-resolution camera over a fragile tether from a moving mouse, is the presence of corrupted frames. These are typically blank frames or distorted, noisy frames. We use a spike detection algorithm described for the detection of extracellular unit activity to detect such images based on the fluctuation in the temporal difference of mean image intensity Ī (Quiroga et al., 2004). Frames exceeding the threshold *thr* are replaced with the preceding frame.

$$\mathrm{thr}=5\cdot \text{median}(\frac{\left|\Delta Ī\right|}{0.6745})$$

Due to vignetting from the used GRIN lenses, fluorescence intensity drops strongly toward the image borders. This static drop presents a challenge for motion correction and other later used algorithms, which is why we implemented a flat-field estimation and correction into CAVE. This flat field correction is done by dividing each frame by the heavily blurred mean image of the time series.

#### Image Alignment

Movement artifacts are one of the main challenges in head-mounted calcium imaging in freely moving animals. In contrast to head-fixed two-photon preparations, the weight and inertia of camera and tether exacerbate such artifacts. Moreover, single-photon imaging leads to much lower image contrast and resolution compared to two-photon imaging, hindering the removal of the artifacts. CAVE offers two different image alignment: subpixel registration (Hotchkiss et al., 1989) and Lucas–Kanade registration (Baker and Matthews, 2003). We found the Lucas–Kanade algorithm to work best for data containing visible local features, such as blood vessels. If such features are missing, subpixel registration is often able to still achieve acceptable results. Image alignment can be performed using a selected region in the image (e.g., a blood vessel) or the full preceding frame. The latter option is particularly helpful if constant features are missing but activity levels are high. In this case, the slow decay of GCaMP fluorescence will allow stabilization of the time series across subsequent frames. Following movement correction, the time series is cropped utilizing the largest shift values to avoid edge artifacts.

#### ΔF/F Calculation

In order to obtain the fluorescence change over baseline, we use standard Δ*F*/*F* calculation. The fluorescence change is calculated by taking the fluorescence intensity of each individual image (*F*_i_), subtracting the mean image across time 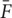, and lastly dividing by the mean image (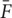):

$$\Delta F/F=\frac{F_{\text{i}}-\bar{F}}{\bar{F}}$$

#### Maximum Intensity Projection

Typically, the region of each cell is brightest during a calcium event. This feature can be used to visually detect cells by using a maximum intensity projection image of the whole time series. Following the previous processing steps, this image is displayed, weighted by the standard deviation of each pixel, to help the user in defining cell ROIs.

#### Cell Detection

Unsurprisingly, it is impossible to manually detect the dozens, hundreds, or even thousands of cells commonly encountered in head-mounted calcium imaging preparations. To detect large numbers of cells at once, we implemented the automatic cell detection via principal and independent component analysis (ICA/PCA) as described previously (Mitra and Pesaran, 1999; Mukamel et al., 2009; Ghosh et al., 2011). ROIs outside a preset size window and roundness value are removed (default: 30–300 pixels, roundness metric below 0.6). Overlapping ROIs with above 30% shared area are merged. Furthermore, double-assignments are merged to a single component when there is an overlap above 30. This automatic detection can be complemented or refined by manual selection or removal of cell ROIs. Further ROI refinement is achieved by excluding ROIs with too low fluorescence and merging highly correlated neighboring ROIs (*r* > 0.8). Through the provided configuration file, these and all other presets can be optimized for each use case.

#### Calcium Activity Deconvolution

**FIGURE 3:**
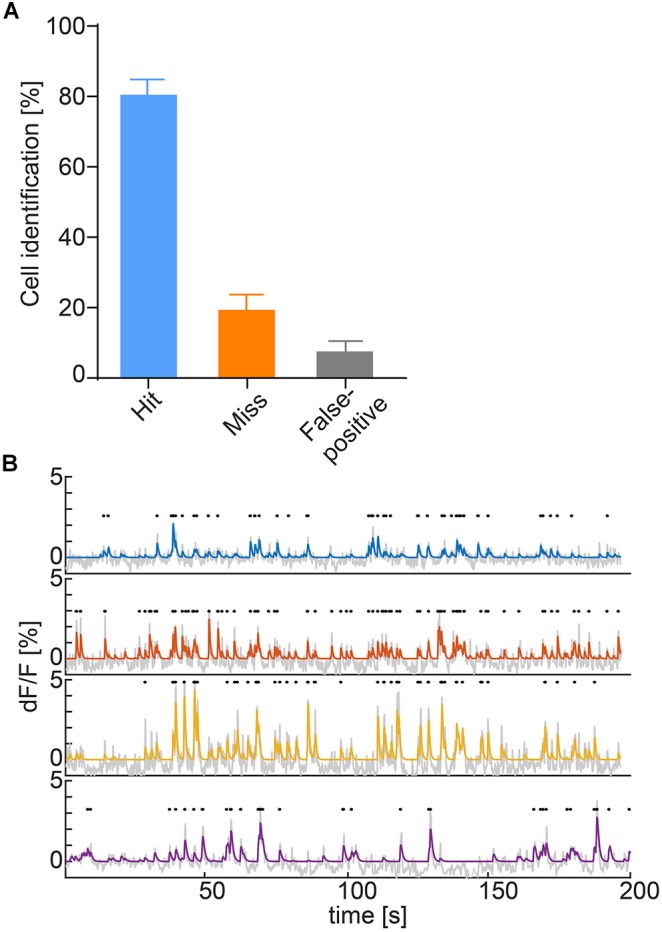
**(A)** Hit (ROI correctly identified automatically), miss (ROI not identified automatically) and false alarm (ROI falsely identified as cell) rates of ROI auto-detection. Data represented as mean ± SEM, *n* = 6. **(B)** Deconvolved calcium traces of four exemplary cells (blue, orange, yellow, and purple), underlying raw traces (gray) and detected calcium transients (black dots).

To obtain identifiable calcium transients from the raw fluorescence traces, deconvolution after neuropil correction is used. The neuropil is defined as a 20-μm ring surrounding the ROI (Kerlin et al., 2010; Chen et al., 2013; Dana et al., 2014; Pinto and Dan, 2015) and the raw values of the neuropil are multiplied by a factor of *r* = 0.7 according to previous research (Chen et al., 2013; Dana et al., 2014). To accommodate for the various imaging systems used, the user can either input a scaling value or select from a range of presets for common systems. Following neuropil correction, the trace is detrended to remove bleaching. In the final step, the fluorescence traces are deconvolved using the OASIS algorithm with a second degree auto-regressive model (Friedrich et al., 2017; Giovannucci et al., 2017; Figure 3B).

#### Animal Tracking

**FIGURE 4:**
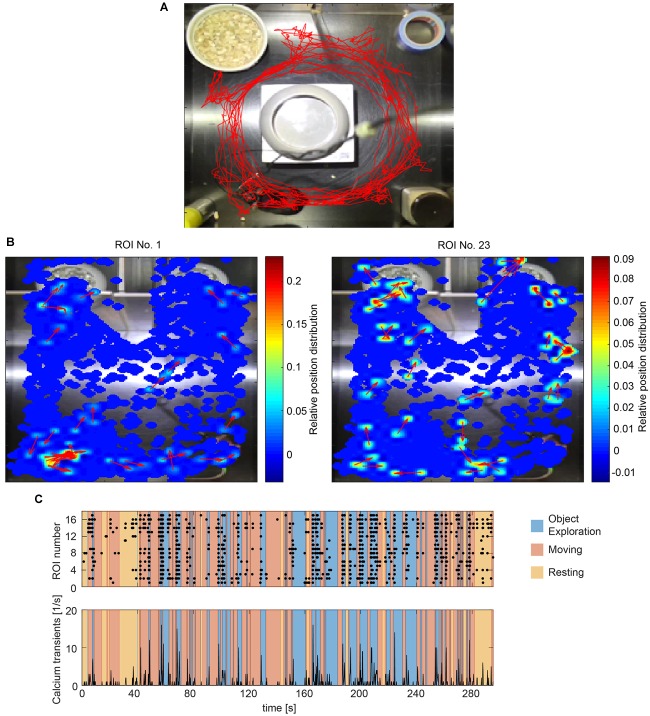
Correlating calcium activity and behavior. **(A)** Tracking a mouse with one color marker (red trace). **(B)** Heatmaps showing calcium transients in relation to the spatial location of the animal for two exemplary cells (ROI 2 and 15). The momentary orientation of the animal is indicated with an arrow during each detected calcium transient. **(C)** Raster plot of calcium events (black dots) and accumulated calcium event rates of all detected cells during one imaging session. Different behaviors are color coded.

Animal tracking is based on the detection of color markers in the image (sticker, LED, colored dye). This method is especially robust against artifacts from the camera tether, which are unavoidable in head-mounted calcium imaging. The use of two markers per animal further allows orientation detection. To extract the markers in the video, the user can manually select regions in HSV color space or use a number of presets. The marker detection algorithm then estimates the center of each colored spot and returns the coordinates of the animal over time (Figure 4A). The quality of the tracking is automatically assessed and the user alerted to untracked frames.

#### Spatial Activity Distribution

To match the location of the mouse with the recorded cell activity, CAVE stores the location of the animal at each time point a calcium transient was detected. A heatmap can then be computed to visualize the dependence of cell activity and spatial location. This feature is especially useful for studying place fields and similar phenomena (Figure 4B).

#### Behavior Annotation

Calcium ActiVity Explorer supports multiple user-definable tracked behaviors. Individual epochs of each behavior are annotated interactively during the course of the behavioral video. After behavior annotation, calcium activity can be analyzed in relation to the behavioral epochs. Raster plots, calcium events rates, and normalized activity plots are supported by default (Figure 4C). For advanced analysis, all results and the raw as well as deconvolved traces are automatically saved and exported.

#### Calcium Activity Sweeps

Following behavior annotation or import of external events, calcium traces can be aligned to different triggers and plotted (Figure 6A). A sweep plot is generated showing individual repetitions in stacked rows, as well as a mean trace below, outlined with the standard error across trials.

## Results

To demonstrate the application of CAVE in the analysis of common types of imaging experiments, we imaged and analyzed data from both a cortical region (primary somatosensory cortex, S1) and a hippocampal region (DG).

### Temporal Evolution of Activity and Cell Statistics

**FIGURE 5:**
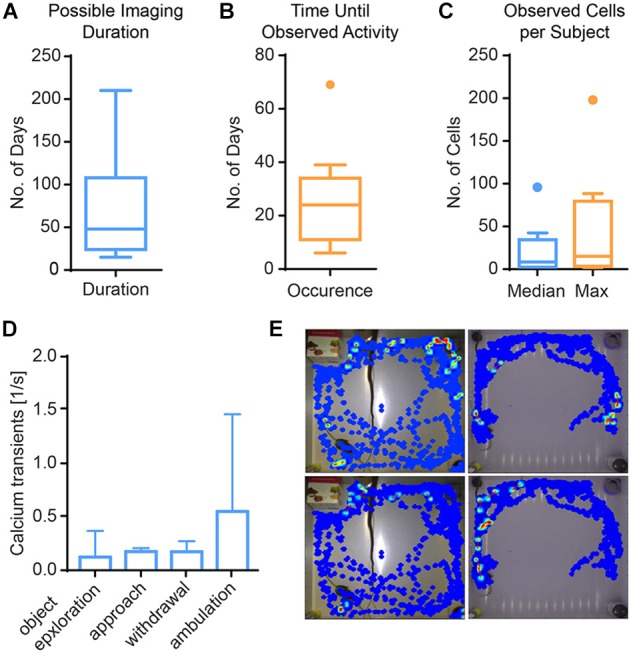
Results from imaging in mouse hippocampus. **(A)** Calcium activity was observable for 67 days on average (Tukey boxplot, *n* = 11). **(B)** First calcium activity occurred after a median of 25 days (Tukey boxplot, *n* = 11). **(C)** Across mice, the median number of cells was 8, and the median of the maximal cell count 15 (Tukey boxplot, *n* = 11). **(D)** Calcium activity is higher during ambulation (traveling movements) than other behaviors, albeit not significantly. Data presented as median ± range, *n* = 3. **(E)** Exemplary heat maps showing calcium transients of four different cells from two mice (left vs. right columns) in relation to their spatial location.

In single-photon imaging through GRIN lenses, a long time span typically passes from lens implantation until clearly identifiable cells become visible. We therefore characterized the time from implantation to visible activity in the hippocampus (DG and CA1, Figure 5). The median time to onset of detectable single cell activity was approximately 1 month (minimum: 6 days, maximum 69 days). Typically, activity persisted for approximately 2 months, with some preparations lasting only a few weeks and some for more than half a year. During the time of visible activity, the median number of identified cells across animals was 8. Within individual animals, the median number of cells across time ranged from 1 to 96. The maximal number of cells per animal ranged from 2 to 198 cells. Out of 25 animals imaged, 11 animals did not show any discernable activity at all.

### Relationship of Cell Activity and Behavior in Dentate Gyrus and Somatosensory Cortex

**FIGURE 6:**
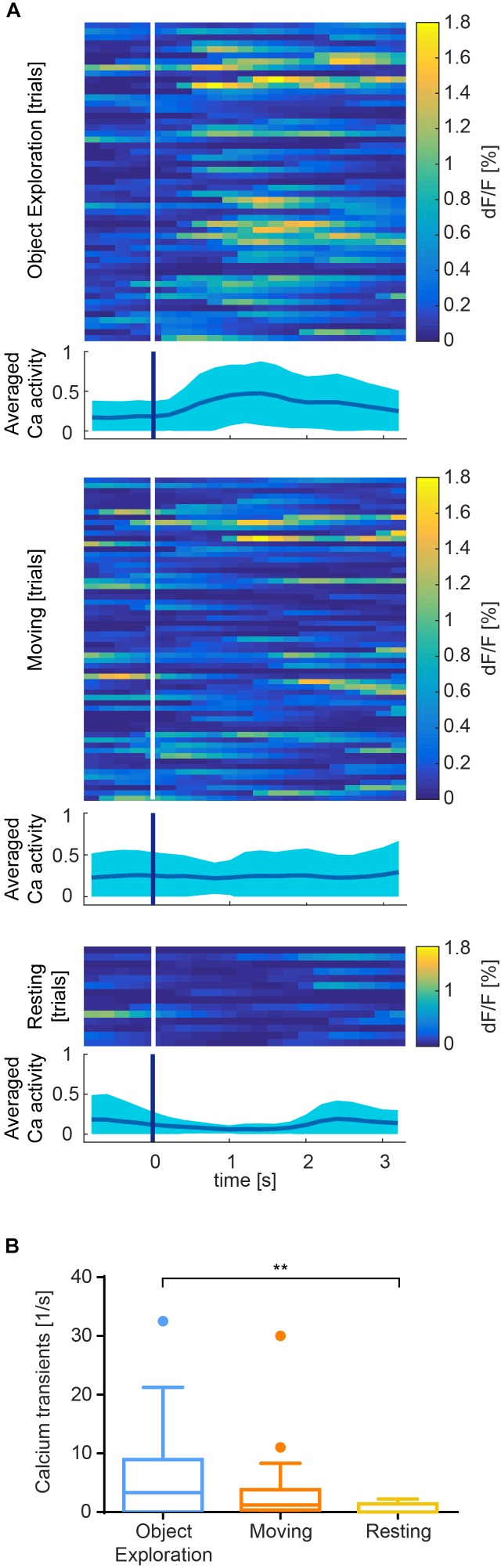
Exemplary results recorded from somatosensory cortex of one session. **(A)** Color raster of fluorescence signal (*dF*/*F*) from episodes of object exploration (*n* = 53), moving (*n* = 62), and resting (*n* = 14). Each row represents mean activity across all ROIs for one trial. Trials are stacked vertically. Calcium activity is higher after onset (white/blue bar) of exploration than moving or resting, which is summarized in an averaged plot below each color raster as mean ± SEM. **(B)** Event rate is higher for object exploration than for moving and significantly higher than resting behavior (*p* < 0.01). Mean activity of 17 identified cells during episodes of behavior.

To showcase the use of CAVE for in-depth analysis of individual sessions and group analysis, we analyzed a set of hippocampus imaging sessions (DG) in three mice and an exemplary somatosensory cortex imaging session in one mouse and (Figure 6).

In the DG, we analyzed the imaging session exhibiting the highest cell count within the first week after onset of activity in three mice. We found a trend of elevated calcium activity particularly during ambulation (traveling movement, Figures 5D,E). The integrated analysis of calcium imaging data and behavioral data in CAVE allows correlating calcium activity and spatial position (Figure 5E).

In S1, we found that calcium transients were significantly more common during object exploration compared to resting behavior (Figure 6B, *p* < 0.01, Kruskal–Wallis non-parametric ANOVA, Dunn’s correction for multiple comparisons). Activity across all ROIs during object exploration was also elevated compared to traveling movement, although not significantly. Notably, the onset of cell activity typically lagged the onset of object exploration (Figure 6A).

### Computational Performance of CAVE

**FIGURE 7:**
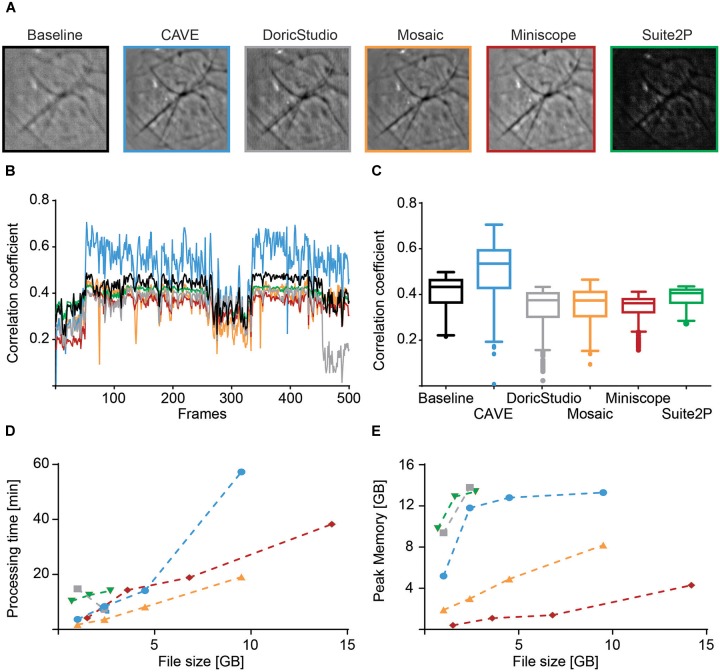
Performance comparison of different calcium imaging tools. **(A)** Mean image before alignment (baseline) and after alignment with different toolboxes. **(B)** Spatial Pearson’s correlation coefficient of each frame in respect to their mean image for baseline (black) and post-alignment mean image for CAVE (blue), DoricStudio (gray), Mosaic (orange), Miniscope Analysis (red), and Suite2P (green) for a representative dataset of 500 frames. **(C)** Boxplots of time-averaged correlation coefficients for the various tools and the same dataset. **(D)** Total processing times for different tools over various sizes of datasets. Suite2P and DoricStudio failed to load larger datasets on the test PC. **(E)** Peak memory load for different tools over various sizes of datasets.

We characterized image alignment performance using mean images as well as spatial correlation of individual frames to post-alignment mean images. CAVE calculated well-aligned mean images (Figure 7A) and showed high correlation of individual frames to the mean frame after alignment (Figure 7B). In total, CAVE had the highest median correlation coefficient of all tested tools (Figure 7C, non-parametric ANOVA (Friedman test) *p* < 0.0001, Dunn’s multiple comparisons post hoc test of CAVE versus any other tool was highly significant *p* < 0.001).

Processing time was shortest for Inscopix Mosaic. CAVE showed medium speeds but was generally faster than Suite2p, a standard in the field (Figure 7D). Similar to Doric Studio, Suite2P failed to load larger data sets on our PC, which is why some values could not be determined for these tools. Despite high-pass filtering, Suite2P failed to correctly identify any cells in our test data.

Peak memory load was average, with Suite2P and Doric Studio requiring more memory and Mosaic and Miniscope Analysis requiring less (Figure 7E).

## Discussion

We developed CAVE as user friendly software, intuitive to novice users but incorporating detailed options for advanced users. CAVE offers streamlined processing of large single-photon calcium imaging datasets from head-mounted microscopes in a GUI. CAVE contains standard ROI-based calcium imaging analysis functions along with features tailored to the specific demands of data obtained from head-mounted microscopes. Specifically, it can compensate for movement artifacts in the typically low-contrast images and correct for optical aberrations and imperfections. Together with its integrated behavioral analysis, CAVE offers a unique set of features not previously found in an open source toolbox.

The calcium signal analysis workflow starts with a preprocessing step that corrects optical imperfections and stabilizes the images. We implemented two alternative stabilization algorithms, Lucas–Kanade and subpixel registration. Both algorithms can be used with a static reference frame or dynamic reference frames. The latter offers stabilization even in the complete absence of static contrasting objects (e.g., blood vessels) in the frame, based purely on the ongoing calcium activity. This approach does, however, require the presence of active cells during the whole imaging session. Using the Lucas–Kanade algorithm, CAVE performed well in comparison to other available tools as measured by correlating individual frames to the mean image. Post-alignment mean images further confirm successful alignment. Nonetheless, exact performance of alignment critically depends on the presence of contrasting features such as vessels and visible cells. As a result, alignment performance will vary with the specific data used.

For automatic ROI extraction, we rely on a PCA/ICA approach. Other algorithms have been proposed (Maruyama et al., 2014; Mohammed et al., 2016; Pnevmatikakis et al., 2016; Zhou et al., 2018), and can be easily implemented due to the open nature of CAVE. PCA/ICA is already a robust algorithm working well for the cell counts found in our data. Given the approximately 400 μm field of view of the microscope used for testing, we did not encounter situations where PCA/ICA did not yield reasonable ROIs. Compared to manual extraction, our implementation of the algorithm yields good sensitivity and specificity (Figure 3A), but might be improved with advanced algorithms (Pnevmatikakis et al., 2016; Zhou et al., 2018). One inherent property of the PCA/ICA algorithm is that if the number of ROIs is grossly overestimated initially, cells are incorrectly split into sub-ROIs. While after a few imaging sessions it is clear to the user how many ROIs can be expected, CAVE reduces this behavior by automatically merging highly correlated, neighboring ROIs. Since CAVE provides comprehensive manual auditing capabilities for ROI definition, missed or falsely defined ROIs can be added and removed later. This combined approach therefore provides a high data quality.

Calcium ActiVity Explorer does not only provide the user with raw intensity values of individual ROIs but offers deconvolution of these traces to reconstruct individual calcium transients. We used the OASIS algorithm described previously (Friedrich et al., 2017) and found it to provide excellent results without being computationally expensive. However, the inference of actual neuronal discharges from calcium imaging data is non-trivial (Vogelstein et al., 2010). This shortcoming of calcium imaging should be kept in mind during data interpretation.

One feature not supported by CAVE in its current form is tracking of cells across sessions. Such tracking is difficult and might not always be achievable in a given preparation using current single-photon technology. The position of the lens shifts slightly over time and dynamic processes alter the tissue in front of the lens. It is typical that new cells appear for every new imaging session and others disappear. Whether a cell detected in a similar position in the following session is indeed a cell from the previous session is therefore often not clear. If tracking is necessary in a given experiment, additional algorithms and tools can be used (Sheintuch et al., 2017). To further enhance longitudinal tracking performance, it might be beneficial to gather additional information from the sample. For example, a second fluorescent marker could be fused to GCaMP (Cho et al., 2017) and imaged in a second wavelength band. Potentially, it might also be possible to obtain depth information about cells, either by varying the focus (e.g., through electrically tunable lenses), gathering stereoscopic information from two viewing angles onto one or two image sensors, or patterned illumination.

In regard to the analysis of behavior we rely on standard color tracking as described previously (Maghsoudi et al., 2018). The main advantage of this strategy is that it is robust against interference from the cables of head-mounted microscopes in the image. In addition, the orientation of the animal can be easily reconstructed from the vector connecting the two colored spots. As shown in the results, the approach worked well in the investigated scenarios.

We tested CAVE in two exemplary imaging scenarios: hippocampal imaging and imaging of sensory neocortex (S1). As expected, in S1, we found time locked activity to the onset of object exploration. In hippocampus, we found highest activity during running, which is in line with previous findings (Bender et al., 2015; López Ruiz et al., 2015). However, we did not find any clear place fields in the generally sparse activity. Either the imaged cells did not respond strongly to a preferred spatial location, or our imaging sessions were too short to map such fields. In general, activity levels were sparse, with many cells firing action potentials just once or twice per minute. Such cells can be reliably imaged with *in vivo* calcium imaging but might be impossible to cluster from electrophysiological recordings.

In addition to analysis of hippocampal activity, we also measured the time until active cells became visible and for how long they could be tracked in our preparation. One important, but little discussed, aspect of GRIN lens based single-photon imaging is the long duration until it becomes obvious whether a particular animal will show activity and this activity can be imaged. Approximately 4 weeks are necessary to determine whether a particular preparation is successful, a time span, which might limit the usefulness of such imaging for anything but non-time critical imaging in adult mice. However, after onset of activity, preparations can typically be imaged for a long time. Another aspect important for planning imaging experiments is the limited yield of cells in many individuals. While hundreds of cells were observed in some mice, most showed much lower numbers. Depending on the structure, the yield is most likely determined by the accuracy of stereotactic placement. It is critical that the distance between lens tip and cell of interest stays in the range of 200 μm for optical reasons. This translates into different levels of necessary stereotactic accuracy depending on brain area. Live imaging during implantation might be beneficial, although we did not see any benefits of it in our experiments.

In summary, CAVE is a novel, open, user-friendly GUI-based tool for the analysis of large single-photon imaging datasets from head-mounted miniature microscopes, combining analysis of calcium imaging and behavioral data.

## Data Availability

The raw data supporting the conclusions of this manuscript will be made available by the authors, without undue reservation, to any qualified researcher.

## Author Contributions

JT and ML designed the foundation of the software while JT, ML, KJ, and H-JH designed the experiments. JT programmed the GUI while ML and MB tested the GUI. JT and MB performed the experiments. JT and ML analyzed the data and prepared the figures. ML, JT, and FO wrote the manuscript.

## Conflict of Interest Statement

The authors declare that the research was conducted in the absence of any commercial or financial relationships that could be construed as a potential conflict of interest.
